# Draft Genome Sequence of the Iron-Oxidizing Acidophile *Leptospirillum ferriphilum* Type Strain DSM 14647

**DOI:** 10.1128/genomeA.01153-14

**Published:** 2014-11-06

**Authors:** Juan Pablo Cárdenas, Marcelo Lazcano, Francisco J. Ossandon, Melissa Corbett, David S. Holmes, Elizabeth Watkin

**Affiliations:** a Fundación Ciência & Vida, Santiago, Chile; b Facultad de Ciencias Biológicas, Universidad Andres, Bello, Santiago, Chile; c School of Biomedical Sciences, Curtin University, Perth, Australia

## Abstract

The genomic features of the *Leptospirillum ferriphilum* type strain DSM 14647 are described here. An analysis of the predicted genes enriches our knowledge of the molecular basis of iron oxidation, improves our understanding of its role in industrial bioleaching, and suggests how it is adapted to live at extremely low pH.

## GENOME ANNOUNCEMENT

*Leptospirillum ferriphilum* is a Gram-negative chemolithoautotrophic bacterium, consistently isolated from metal-rich, mesophilic (25 to 40°C), and acidic environments (pH 1.3 to 2.0) where iron-bearing minerals are exposed to oxygen and water. Although the morphology of *L. ferriphilum* is variable, it is usually described as consisting of small (0.3 to 0.9 µm), vibroid- to spiral-shaped cells, with spirals of 3 to 12 cells. It possesses a single polar flagellum and is obligately chemolithotrophic, utilizing CO_2_ as a carbon source. Its energy requirements are met by the aerobic oxidation of ferrous iron but not sulfur, and within industrial biooxidation tanks that possess high redox potentials, *L. ferriphilum* becomes the dominant iron oxidizer (1). The genomic sequencing of the *L. ferriphilum* type strain DSM 14647 provides important information regarding its bioleaching capabilities. The type strain (DSM 14647) was originally isolated from Peru (2).

The genome of *L. ferriphilum* strain DSM 14647^T^ was sequenced using the Roche GS-FLX 454 sequencing platform and assembled using Newbler (version 2.6), with *L. ferriphilum* ML-04 (accession no. CP002919) serving as a reference genome. The draft genome size is 2.4 Mb, with a median coverage depth of 23-fold and an average G+C content of 54.2%. It contains 20 large (>1,000 bp) contigs, with an *N*_50_ of 274,009, and 15 smaller contigs. The genes and RNA features were identified using RAST (3). The draft genome annotation predicts 44 tRNA sequences and 2,726 protein-coding genes, 53.5% of which were assigned putative functions (4).

The genome has genes predicted to encode enzymes of the reverse tricarboxylic acid (TCA) cycle (5). Nif genes, involved in nitrogen fixation, were not detected in this genome; however, it exhibits genes for ammonium assimilation. It also has genes that encode electron transfer proteins proposed to be involved in iron oxidation, including cytochrome_572_, cytochrome_579_, and 2 copies of the terminal electron acceptor *cbb3* complex (6). Genes involved in flagella biosynthesis and chemotaxis were also detected. As described previously for other members of the genus (7), it has genes encoding enzymes for the ectoine and trehalose biosynthetic pathways. It also has genes predicted to be involved in heavy metal efflux (*czcABC*), arsenic resistance (*arsBC*), and mercury resistance (*merA*). The genome contains genes potentially encoding several components of a type IV secretion system involved in conjugative DNA transfer (8) and genes for a clustered regularly interspaced short palindromic repeat (CRISPR)-Cas system putatively involved in antiviral resistance (9).

### Nucleotide sequence accession number.

This whole-genome shotgun project has been deposited at DDBJ/EMBL/GenBank under the accession no. JPGK00000000.
